# Effects of pH-shifting and ultrasound on the structural and emulsifying properties of peanut globulin fractions

**DOI:** 10.1016/j.fochx.2024.101390

**Published:** 2024-04-13

**Authors:** Lin Chen, Shao-Bing Zhang, Shu-Yan Zhang

**Affiliations:** College of Food Science and Engineering, Henan University of Technology, Zhengzhou, Henan Province 450001, China

**Keywords:** Peanut proteins, Arachin, Conarachin, pH-shifting, Ultrasonication, Emulsifying performance

## Abstract

This work investigated and compared the structural and emulsifying properties of peanut globulin fractions (conarachin and arachin) after ultrasonication (US) and pH_2.5_-shifting treatments, singly and in combination. Results showed that pH_2.5_-shifting was more effective in degrading peanut protein subunits and unfolding their structures than US treatment. Conarachin tended to aggregate during US and pH_2.5_-shifting treatments possibly due to higher free sulfhydryl content, while high molecular weight arachin tended to disaggregate during these treatments. pH_2.5_-shifting or US+pH_2.5_-shifting treatments significantly increased the surface hydrophobicity of conarachin (from 72 to 314) and arachin (from 336 to 888), which may be responsible for the enhancement of protein emulsifying activity. All treatments significantly improved the physical stability of arachin-stabilized emulsions with higher absolute potentials but lowered that of conarachin-stabilized emulsions. However, pH_2.5_-shifting or US+pH_2.5_-shifting treatments could improve the stability of conarachin-stabilized emulsions in the presence of salts.

## Highlights

• pH-shifting was more effective in modifying conarachin and arachin than ultrasound.
• Both treatments promoted conarachin aggregation and arachin disaggregation.
• pH-shifting improved the emulsifying activity of both conarachin and arachin.
• Conarachin- and arachin-stabilized emulsions have opposite changes in stability.

## 1 Introduction

Peanut proteins are among the most important plant proteins in the world and contain two major globulins: arachin and conarachin. Arachin with a molecular weight of ∼350 kDa comprises three acidic subunits and three basic subunits. Conarachin with a molecular weight of ∼180 kDa contains three identical subunits. Conarachin has a looser tertiary conformation and lower thermal stability compared with arachin (Zhao, Liu, Su, & Wang, 2017). The contents of methionine, lysine and cysteine in conarachin were 3 times, 2 times and 2 times that of arachin (Chiou, 1990). The ratio of arachin to conarachin varies from 0.80 to 1.68 depending on the cultivar of peanut (Du, Wang, Liu, Wang, & Liu, 2013).

Native peanut proteins generally show poor functionalities because of their compact conformation. To enhance the functional properties of peanut protein isolate (PPI), many physical and chemical modification methods have been reported, such as enzymatic hydrolysis (Zhang, Song, Chang, Wang, & Meng, 2022), ultrasonication (US) (Zhang et al., 2014), high-pressure processing (He et al., 2014), pH-shifting (Li et al., 2020; Wang et al., 2020), and transglutaminase cross-linking (Zhang, Wang, Li, & Yan, 2020). Compared with other modification methods, the biggest advantage of pH-shifting is that it is easy to operate. pH-shifting is based on the principle of exposing a protein to an extreme acidic or alkaline pH to induce structural unfolding, followed by adjusting the pH back to neutral to allow protein refolding (Jiang, Wang, & Xiong, 2018). Li et al. (2020) studied the effects of acid and alkaline pH-shifting on the structural and gelling properties of PPI. After PPI was exposed to pH 10.0 for 1 h, its gelling properties were significantly improved, while its particle size decreased and its solubility, free sulfhydryl group content and surface hydrophobicity increased. However, PPI lost its gelling ability when exposed to pH 2.0 and 12.0 due to the formation of large protein aggregates.

US is another rapidly developing environmentally friendly technology that can alter protein structures and functionalities mainly by acoustic cavitation (Yu et al., 2023). To improve the modification effects of pH-shifting alone, US and pH-shifting combined treatments have been carried out in many studies. For example, pH-shifting and US combined treatments significantly improved the solubility and surface hydrophobicity of pea protein isolate (Jiang et al., 2017). The combination of US and alkaline conditions could potentially alter the secondary structure of WPI and enhance its emulsifying properties (Gao et al., 2019). Recently, Jiang, Zhang, Julian McClements, Liu, and Liu (2022) prepared pea protein-inulin conjugates via the Maillard reaction using a combination of US and pH-shift treatments. They found the conjugate-stabilized algae oil emulsion had better stability. For peanut proteins, it was reported that US under acidic conditions (pH 3.0) can markedly promote subunit dissociation and unfolding of PPI (Huang, Zhang, Li, Ji & Ma, 2016). Although some structural and functional changes of PPI induced by pH-shifting and US treatments have been revealed by the above work, the physicochemical properties of PPI globulin fractions (conarachin and arachin) when subjected to these treatments have rarely been investigated. Due to the vast differences in the molecular size and other structural properties of arachin and conarachin, they may experience different changes in these treatments.

In this work, conarachin and arachin were subjected to pH-shifting, US, and US + pH_2.5_-shifting treatments, respectively. The structural and emulsifying properties of the two peanut globulin fractions before and after these treatments were investigated and compared. The structure-function relationships of peanut proteins were discussed. The results may lead to a better understanding of the changes in the physiochemical properties of peanut proteins induced by pH-shifting and US treatments and provide a theoretical basis for the application of peanut protein fractions in the food industry.

## 2 Materials and methods

### 2.1 Materials

Peanut seeds and soybean oil were purchased from the local market. 8-Anilinonaphthalene-sulfonic acid (ANS) was obtained from Yuanye Bio-Technology Co., Ltd. (Shanghai, China). 5,5’-Dithiobis-(2-nitrobenzoic acid) (DTNB) and commercial precast gels (P1200, 5% acrylamide for the stacking gel and 12% acrylamide for the separating gel) were purchased from Solarbio Science & Technology Co., Ltd. (Shanghai, China). All other reagents were of analytical grade.

### 2.2 Aqueous extraction of peanut protein isolate (PPI) from peanuts

Unpeeled peanut seeds (300 g) were put into an all-purpose blender and ground for 3 min. Then, the peanut paste was dissolved in 6 times distilled water, and the pH was adjusted to 10.0 with 2 M NaOH. The suspension was placed in a 50 °C water bath shaker (SHZ-88, Jiangsu Instrument Co., Ltd., Jingyi, China) with an oscillation speed of 150 rpm for 30 min, and the upper oil layer, milk layer, and lower sediment were removed by centrifuging the mixture for 15 min at 4000 rpm. The pH of the supernatant was adjusted to 4.5 with 2 M HCl followed by centrifugation (4000 rpm, 10 min) to obtain the precipitate. The precipitate was then washed twice with distilled water. The obtained sample (PPI) was freeze-dried and stored in a desiccator.

### 2.3 Extraction of arachin and conarachin from PPI

PPI was mixed with 3-fold phosphate buffer solution (0.3 mol/L, pH 7.5), stirred at room temperature for 1 h, and then centrifuged (4000 r/min, 15 min), and the insoluble substance was discarded. The supernatant was treated in two ways to obtain arachin and conarachin. (1) Ammonium sulfate was added to the supernatant until its saturation reached 40%. The solution was stirred evenly and then centrifuged (2000 r/min, 10 min). The obtained precipitate (arachin) was dialyzed (48 h) to remove ammonium sulfate and then freeze-dried (protein content: 82.05 ± 0.15%, N × 5.46). (2) Ammonium sulfate was added to the supernatant until it reached 60% saturation. The solution was stirred well and subsequently centrifuged at 4 °C (8000 r/min, 20 min) to remove arachin. Then, ammonium sulfate was added to the supernatant until it reached 85% saturation. This solution was stirred and subsequently centrifuged as described above. The precipitate was dialyzed (48 h) to remove ammonium sulfate and then freeze-dried to obtain conarachin (protein content: 86.23 ± 0.28%, N × 5.46).

### 2.4 pH-shifting and ultrasonic treatments

Protein suspensions (1%, *w*/*v*) were prepared by dispersing the protein samples in distilled water into a 200-mL beaker. The pH of the dispersions was adjusted to 8.0 with 2 M NaOH and subsequently stirred gently at room temperature until they were fully dissolved. The protein dispersions were subjected to three treatments: pH-shifting, US, and US + pH_2.5_-shifting. In pH-shifting alone treatments, the pH of the samples was adjusted to 2.5 with 2 M HCl. The samples were then stored at room temperature for 2 h and subsequently neutralized to pH 8.0 with 2 M NaOH.

In treatments with US alone, the protein dispersions were treated with an ultrasonic cell disintegrator (Scientz-IID, NingBo Scientz Biotechnology Co. Ltd., Ningbo, China) with a titanium probe (0.636 cm diameter). The solution temperature was controlled to be ≤35 °C in an ice-water bath. Samples (25 mL) were treated under different levels of power output (100 and 600 W) at different times (5 and 20 min) (pulse durations of on-time 3 s and off-time 2 s).

In US + pH_2.5_-shifting treatments, the pH of the samples was adjusted to 2.5 with 2 M HCl. The samples were then stored at room temperature for 1 h followed by US treatments under different levels of power output (100 and 600 W) at different times (5 and 20 min). Next, the samples were stored at room temperature for 1 h and subsequently neutralized to pH 8.0 with 2 M NaOH. These treated samples were freeze-dried and stored in a desiccator.

### 2.5 Protein structural properties

#### 2.5.1 SDS-polyacrylamide gel electrophoresis (SDS–PAGE)

SDS-PAGE was performed according to the method of Zhang and Lu (2015) with modifications. Protein suspensions (3 mg/mL) were prepared by dispersing the protein samples in a sample buffer [10% (*v*/v) Tris-HCl buffer (pH 8.0) containing 0.8% (*w*/*v*) SDS, 4% (v/v) glycerol, 10% (v/v) β-mercaptoethanol (not used when the nonreducing electrophoresis was performed) and 0.004% (w/v) bromophenol blue]. The protein suspensions (10 μL) were then mixed with an equal volume of sample buffer, and the mixtures were placed in boiling water for 5 min. The electrophoresis was then run at 80 mA in a stacking gel and at 120 mA in a separating gel until the tracking dye reached the bottom of the gel. After electrophoresis, the gel was immediately dyed with Coomassie Blue R250 for 2 h.

#### 2.5.2 Intrinsic fluorescence emission spectroscopy

The intrinsic fluorescence emission spectra of protein samples were estimated with a fluorescence spectrometer (Cary Eclipse, Agilent Inc., California). Protein suspensions (1 mg/mL) were prepared by dispersing the protein samples in phosphate buffer (pH 8.0, 0.01 mol/L). The fluorescence spectrum was excited at 290 nm, and emission spectra were recorded from 300 to 400 nm. For analysis, the maximum emission wavelength (λ_max_) of the endogenous fluorescence spectrum was recorded.

#### 2.5.3 Surface hydrophobicity

The surface hydrophobicity was determined using 1,8-anilinonaapthalenesulfonate (ANS) as a fluorescent probe according to the method of Zhang and Lu (2015) with minor modifications. Briefly, the protein samples were dissolved in phosphate buffer (pH 8.0, 0.01 mol/L). This protein suspension was then centrifuged at 6000 r/min for 15 min. The protein concentration of the supernatant was determined by the Kjeldahl method (N × 5.46). Then, 4 mL of protein sample solution with various concentrations (0.1–0.6 mg/mL) was mixed well with 20 μL of ANS (8 mmol/L). After 40 s, the relative fluorescence intensities of the ANS–protein conjugates were measured with a fluorescence spectrometer at wavelengths of 380 nm (excitation) and 480 nm (emission). The initial slope of the plot of relative fluorescence intensity versus protein concentration was employed as an index of the protein surface hydrophobicity.

#### 2.5.4 Exposed free SH content

The exposed free SH content of the protein samples was determined using the 5,5′-dithiobis (2-nitrobenzoic acid) (DTNB) reagent according to the method of Zhang, Yan, Jiang, and Ding (2021) with modifications. Ellman's reagent was prepared by dissolving 40 mg of DTNB reagent in 10 mL of Tris-glycine buffer (pH 8.0) containing 0.086 M Tris, 0.09 M glycine and 4 mM EDTA. Protein samples (30 mg) were solubilized in 10 mL of Tris-glycine buffer and centrifuged (6000 rpm, 15 min). The protein concentration of the supernatant was determined by the Kjeldahl method (N × 5.46). Then, 50 μL of Ellman's reagent was added to 3 mL of supernatant and incubated for 1 h at 25 °C. The buffer solution was used as a control sample, and the absorbance of the suspension was read at 412 nm. The SH content was calculated using Eq. (1):

(1) $$\mathrm{SH}\left(\mathrm{\mu mol}/g\right)=\frac{73.53A_{412}}{C}$$

where A_412_ is the absorbance and C is the sample protein concentration (mg/mL).

#### 2.5.5 Particle size

The particle size of the protein samples was measured using a dynamic light scattering analyzer (Zetasizer Nano-ZS90, Malvern Instruments, Worcestershire, UK). The protein samples were sufficiently dispersed in phosphate buffer (pH 8.0, 0.01 mol/L) to obtain protein suspensions (0.1%, *w*/*v*), which were centrifuged at 6000 rpm for 15 min before measurements. All experiments were carried out at room temperature. The average particle size was calculated with the use of computerized instrument processing and Origin software.

### 2.6 Protein functional properties

#### 2.6.1 Solubility

The protein samples were sufficiently dispersed in distilled water to obtain protein suspensions (0.1%, *w*/*v*). The pH was then adjusted to 8.0 with 2 M NaOH. After 2 h of stirring, the dispersions were centrifuged at 4000 rpm for 20 min. The protein content of the supernatants was determined by the Kjeldahl method (N × 5.46). The protein solubility was expressed as grams of soluble protein per 100 g of protein.

#### 2.6.2 Emulsifying activity index (EAI)

The EAI of protein samples was determined according to the method of Zhang et al. (2020) with some modifications. The protein solution (75 mL, 1%, w/v) was mixed with soybean oil (25 mL) and then sheared at high speed (10,000 rpm, 1 min) using a high-speed shearing machine (Model FA25, Fluko Equipment Shanghai Co., Ltd., Shanghai, China). The obtained coarse emulsions were then treated by an ultrasonic cell disintegrator with a titanium probe (0.636-cm diameter). The samples were sonicated (5 min, pulse duration of 2 s and off time of 3 s) at 300 W to prepare the fine emulsions. Aliquots of the fresh fine emulsion (50 μL) were taken from the bottom of the emulsion and added to 5 mL of a 0.1% (*w*/*v*) SDS solution. The absorbance value was immediately measured at 500 nm using a spectrophotometer, with the SDS solution used as a blank. The EAI was calculated according to Eq. (2):

(2) $$\mathrm{EAI}\;\left(m^{2}/g\right)=\frac{2\times 2.303\times \varepsilon \times \mathrm{DF}}{c\times L\times \varphi \times 10000}$$

where ε is the absorbance, c is the protein content (g/mL), φ is the oil volume fraction, L is the light diameter of the cuvette (1 cm), and DF represents the dilution factor.

#### 2.6.3 Physical stability of protein-stabilized emulsions during storage

For the storage experiments, 20 mL of the fine emulsions was added to a glass vial, and 0.05% (w/v) sodium azide was added to prevent the emulsions from spoiling. The emulsions were stored at room temperature for 4 weeks. The changes in the emulsions were periodically observed and recorded by a camera. To investigate the effect of salt addition on the stability of the emulsions, CaCl_2_ was added to the emulsions to achieve a final concentration of 150 mmol/L. The emulsions containing salts were stored and observed as described above.

#### 2.6.4 Centrifugal stability of protein-stabilized emulsions

The centrifugal stability of protein-stabilized emulsions was determined according to the method of Zhang, Zeng, He, and Chen (2012) with minor modifications. The absorbance value at 500 nm of the fresh fine emulsion diluted with 0.1% (*w*/*v*) SDS solution was measured as described above. To evaluate the centrifugal stability, the fresh fine emulsion (10.0 mL) was centrifuged at 3000 r/min for 10 min, and then 50 μL of liquid was taken 2 cm away from the bottom of the centrifuge tube and added to 5.0 mL of 0.1% SDS solution. The absorbance value of the diluted sample solution was measured at 500 nm. The centrifugal stability index was calculated according to Eq. (3):

(3) $$\text{Centrifugal stability index}\;\left(\%\right)=\frac{A_{t}}{A_{0}}\times 100$$

where A_0_ and A_t_ are the absorbance of the emulsions before and after centrifugation, respectively.

#### 2.6.5 ζ-potential of protein-stabilized emulsions

The ζ-potential of protein-stabilized fresh emulsions was measured using a dynamic light scattering analyzer. The emulsions were diluted 100 times with phosphate buffer (pH 8.0, 0.01 mol/L) before the measurements to avoid multiple scattering effects. All experiments were carried out at room temperature. The average ζ-potential was calculated with the use of computerized instrument processing and Origin software.

### 2.7 Statistical analysis

Two different batches of arachin and conarachin were extracted and treated, and all determinations were repeated at least twice. The significant difference (*p* < 0.05) between various samples was analyzed through ANOVA by Duncan's multiple range tests.

## 3 Results and discussion

### 3.1 Subunit compositions

**Fig. 1:**
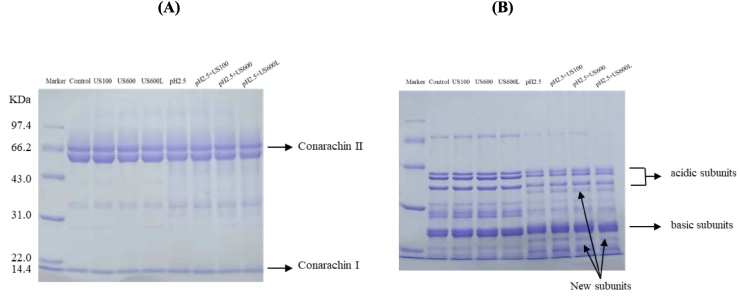
SDS-PAGE profile of conarachin (A) and arachin (B) before and after pH-shifting and ultrasonic treatments. Marker molecular weight range (97.4–14.4 kDa). US: ultrasonication; US100: US treatment (100 W, 5 min); US600: US treatment (600 W, 5 min); US600L: US treatment (600 W, 20 min) (the same below).

To elucidate the changes in the compositions of the conarachin, and arachin subunits induced by US and pH-shifting treatments, electrophoresis was performed under reducing and nonreducing conditions. As shown in Fig. 1, US treatments with different intensities did not alter the electrophoretic patterns of conarachin and arachin, whereas their band brightness levels were significantly decreased after pH_2.5_-shifting or pH_2.5_-shifting+US treatments. At least three new subunit bands appeared on the gel of arachin (Fig. 1B). These results confirmed those reported previously (Zhao, Xin, Zhao, Chen, & Cai, 2014). They found that when the pH value was lower than 3.5, a new 33 kDa band of peanut proteins was produced, and the intensity of the 18 kDa band increased. The cleavage of partial peptide bonds was induced by pH-shifting rather than US, indicating that the former could modify the peanut protein structure to a higher degree. Aspartic acid residues on the peptide chains of several proteins were previously reported to be sensitive to dilute acid (Schultz, Allison, & Grice, 1962). Unlike acidic subunits, the basic subunits of arachin seemed not to be sensitive to the pH-shifting treatment, which might be because basic subunits are usually buried in the interiors of globular proteins (Plietz, Zirwer, Schlesier, Gast, & Damaschun, 1984).

Compared with US treatment alone, US and pH_2.5_-shifting combined treatments did not aggravate the degradation of peanut protein subunits, which is not consistent with the results reported by Huang et al. (2016). In their work, the combination of US and acidic treatments further promoted subunit dissociation of PPI. It should be noted that the extreme pH (3.0) was not readjusted back to neutrality in their work. The difference in acid treatment might explain the inconsistency of the results.

### 3.2 Intrinsic fluorescence and surface hydrophobicity

**Fig. 2:**
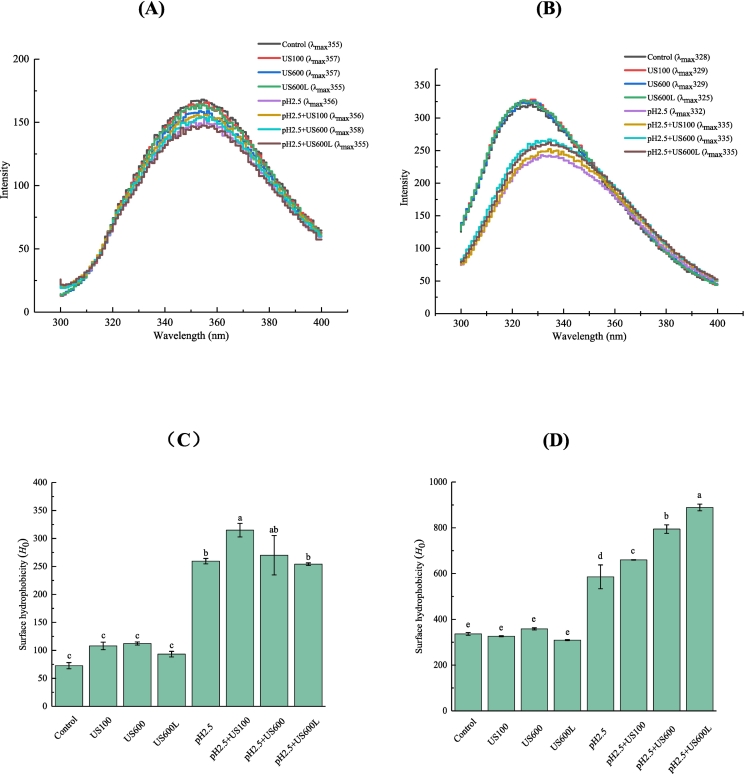
Intrinsic fluorescence and surface hydrophobicity of conarachin (A, C) and arachin (B, D) before and after pH-shifting and ultrasonic treatments. The results are expressed as means ± SD. Bars with different letters indicate significant differences (*p* < 0.05).

To investigate and compare the tertiary conformation of various peanut proteins, intrinsic fluorescence measurements were carried out. The changes in the maximum emission wavelength (λ_max_) of tryptophan reflect its exposure degree to the polar microenvironment. For conarachin (Fig. 2A), neither US nor pH_2.5_-shifting treatments significantly altered its λ_max_, which may be because conarachin originally had a more flexible molecular structure. For arachin (Fig. 2B), US treatment did not significantly change its intrinsic fluorescence, while pH_2.5_-shifting treatment significantly increased its λ_max_ (redshift), suggesting that arachin following pH-shifting treatment could partially unfold due to the increase in electrostatic repulsions. Li et al. (2020) and Huang et al. (2016) reported similar results for PPI. US and pH_2.5_-shifting combined treatments further increased the λ_max_ of arachin, indicating that it was more easily stretched under these treatments.

Surface hydrophobicity (*H*_0_) was analyzed to estimate the degree of protein unfolding. As shown in Fig. 2, pH_2.5_-shifting is more effective than US treatment in improving surface hydrophobicity of conarachin and arachin. The increase in *H*_0_ is generally ascribed to the disruption of the protein tertiary structure and the exposure of hydrophobic groups (Jiang, Chen, & Xiong, 2009). In contrast, Jiang et al. (2017) found that when pea isolate proteins were studied, US treatment improved *H*_0_ better than pH-shifting treatment. The inconsistency of the results indicated that various proteins might have different sensitivity to US or pH-shifting treatments. The *H*_0_ values (336) of untreated arachin were further increased to 888 with US (at higher intensities) and pH_2.5_-shifting combined treatments, which was consistent with the changes in λ_max_. Gao et al. (2019) also reported that the combination of US and pH-shifting treatment resulted in a higher *H*_0_ of WPI than pH-shifting alone treatment. The double effects of physical and chemical modifications likely promoted the exposure of hydrophobic groups to the surface of protein molecules. In contrast, the *H*_0_ values of conarachin were decreased with US (at higher intensities) and pH_2.5_-shifting combined treatments, which may be due to the fact that conarachin underwent partial aggregation under these conditions, thus burying the surface hydrophobic groups into the interior.

### 3.3 Exposed free sulfhydryl content

**Fig. 3:**
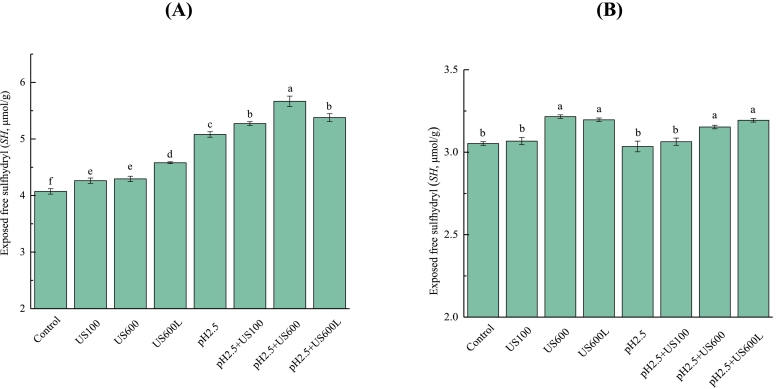
Exposed free sulfhydryl contents of conarachin (A) and arachin (B) before and after pH-shifting and ultrasonic treatments. The results are expressed as means ± SD. Bars with different letters indicate significant differences (*p* < 0.05).

As shown in Fig. 3, untreated conarachin had a significantly higher exposed free sulfhydryl (SH) content than untreated arachin. Both US and pH_2.5_-shifting treatments increased the SH content of conarachin, yet the pH_2.5_-shifting treatment had a more pronounced effect. The combination of US (600 W, 5 min) and pH _2.5_-shifting treatments resulted in the highest SH content of conarachin (Fig. 3A). The unfolding of conarachin results in the exposure of free sulfhydryl groups that are originally located inside the molecules. Li et al. (2020) also reported a significant increase in the SH content of PPI due to pH-shifting at 2.0. However, the SH content of conarachin was deceased with higher intensity US (600 W, 20 min) and pH _2.5_-shifting combined treatments, possibly due to further oxidation of the sulfhydryl groups to disulfide bonds under these conditions. For arachin (Fig. 3B), only US treatments with higher intensities or US and pH-shifting combined treatments slightly increased its SH contents. Since free sulfhydryl plays a major role in disulfide-mediated aggregation of proteins, it is speculated that conarachin may be more likely to aggregate than arachin after their molecular structures are perturbed by US or pH-shifting treatments.

### 3.4 Particle size

**Fig. 4:**
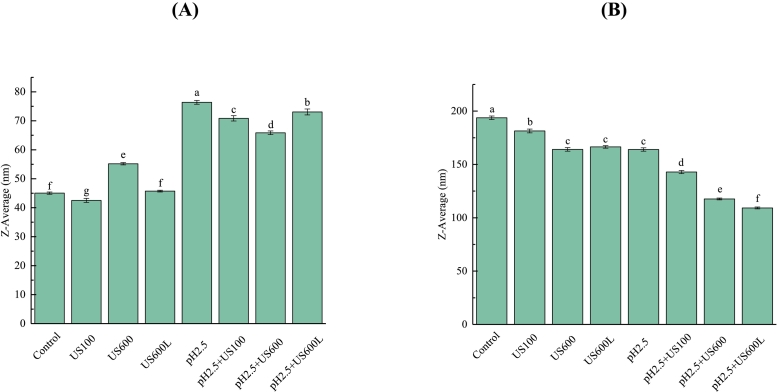
Particle sizes of conarachin (A) and arachin (B) before and after pH-shifting and ultrasonic treatments. The results are expressed as means ± SD. Bars with different letters indicate significant differences (*p* < 0.05).

Figure 4 showed that the particle size of untreated conarachin was markedly smaller than that of untreated arachin. Their particle sizes show opposite patterns of change after US and pH-shifting treatments. For small conarachin (Fig. 4A), both US (600 W, 5 min) and pH _2.5_-shifting treatments significantly increased its particle size. Compared with US treatment, pH_2.5_-shifting increased the particle size of conarachin to a greater extent (from 45 to 77 nm). As discussed above, conarachin had a flexible structure with more exposed free sulfhydryl, which may be responsible for its aggregation during these treatments.

For large arachin with more subunits (Fig. 4B), its particle size gradually decreased with increasing US intensity, an observation in agreement with the studies of peanut proteins (Zhang et al., 2014), soy proteins (Hu et al., 2013), and dairy proteins (O'Sullivan, Arellano, Pichot, & Norton, 2014). This indicates that the physical forces produced by acoustic cavitation can disrupt the interactive forces (noncovalent or/and covalent bonds) between subunits and result in partial dissociation of arachin. Like US treatment, pH_2.5_-shifting significantly decreased the particle size of arachin as well, which may be attributed to not only partial dissociation of arachin but also the subunit degradation induced by acid treatment (Fig. 1B). Additionally, the combination of US and pH_2.5_-shifting treatments markedly reduced the particle size of arachin. Similar results for SPI were reported by Lee et al. (2016). They explained that pH-shifting treatment caused an increase in the surface areas of SPI, and the larger surface areas interacted with ultrasonic waves to produce smaller particle sizes.

### 3.5 Emulsifying activity index (EAI) and solubility

**Fig. 5:**
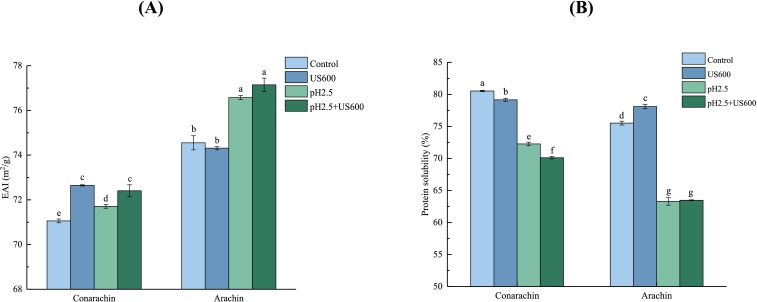
Emulsifying activity index (EAI) (A) and solubility (B) of conarachin and arachin before and after pH-shifting and ultrasonic treatments. The results are expressed as means ± SD. Bars with different letters indicate significant differences (*p* < 0.05).

Based on the above results, the protein samples treated with US (600 W, 5 min), pH _2.5_-shifting (2 h), and US + pH_2.5_-shifting were selected to evaluate their emulsifying properties. Fig. 5A showed that untreated arachin had a higher EAI than untreated conarachin. It is known that solubility and surface hydrophobicity are two important factors affecting the emulsifying properties of proteins. Generally, the higher the value of these two factors, the better the protein emulsification performance. Although the solubility of conarachin was a little higher than that of arachin (Fig. 5B), the surface hydrophobicity of the former was significantly lower as described above. US treatment significantly enhanced the EAI conarachin but did not affect that of arachin. Sun, Zhang, Zhang, Tian, and Chen (2020) pointed out that compared with the EAIs of arachin and conarachin from alkali-soluble and acid precipitation, those from ultrasound-assisted extraction had higher values. pH_2.5_-shifting or US+pH_2.5_-shifting treatments only slightly increased the EAI of conarachin but significantly enhanced the EAI of arachin (Fig. 5A). Although the solubility of conarachin and arachin was remarkably reduced by these corresponding treatments, their surface hydrophobicity was markedly increased. The enhancement of lipophilic capacity of conarachin and arachin promoted rapid protein adsorption at the oil–water interfaces. Jiang et al. (2009) and Huang, Ding, Li, and Ma (2019) reported that the EAI of SPI could be significantly improved by pH-shifting treatment and the combination of ultrasound and acid treatments, respectively.

### 3.6 Emulsion physical stability

**Fig. 6:**
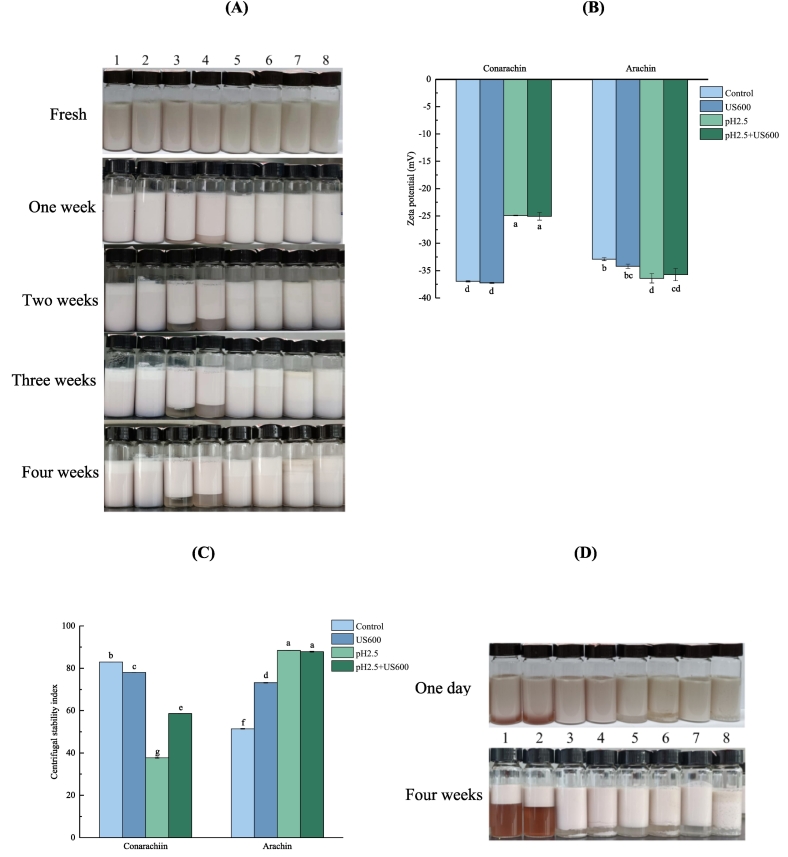
Physical stability of conarachin and arachin before and after pH-shifting and ultrasonic treatments. (A) Storage stability of protein-stabilized emulsions. (B) ζ-potential of protein-stabilized emulsions. (C) Centrifugal stability of protein-stabilized emulsions. (D) Storage stability of protein-stabilized emulsions containing 150 mmol/L CaCl_2_. 1, Conarachin; 2, Conarachin treated with US 600; 3, Conarachin treated with pH 2.5; 4, Conarachin treated with pH 2.5 + US 600; 5, Arachin; 6, Arachin treated with US 600; 7, Arachin treated with pH 2.5; 8, Arachin treated with pH 2.5 + US 600. The results are expressed as means ± SD. Bars with different letters indicate significant differences (*p* < 0.05).

Figure 6A showed that no obvious gravitational separation was observed in all arachin-stabilized emulsions during 4 weeks of storage. The emulsions prepared by untreated or US-treated conarachin exhibited similar stability to arachin emulsions. However, pH_2.5_-shifting or US+pH_2.5_-shifting treatments significantly lowered the stability of conarachin emulsions. The emulsions prepared by the two treated samples showed obvious gravitational separation after storage for only 1 week. By comparison of the ζ-potential of all protein emulsions (Fig. 6B), it was observed that the unstable conarachin emulsions had significantly lower absolute potentials (∼25 mV) than other emulsions. In general, emulsions are more stable when their absolute potential is higher than or equal to 30 mV (Dickinson, 2009). The dramatic decrease in the absolute potential may be associated with the aggregation of conarachin during pH_2.5_-shifting or US+pH_2.5_-shifting treatments. More negatively charged groups were probably packed into the interior of conarachin during these treatments. We have also previously found a correlation between protein aggregation and potential reduction in our studies of PPI (Chen & Zhang, 2023).

To further distinguish the physical stability of protein emulsions, the centrifugal experiments was carried out. Centrifugation can accelerate the changes of the emulsions by applying an appropriate centrifugal force, thus overcoming the shortcoming of long-term storage to judge the stability difference of the emulsions (Zhang et al., 2012). Fig. 6C showed that untreated conarachin emulsion had a higher centrifugal stability coefficient than untreated arachin emulsion. As discussed above, the higher content of exposed free sulfhydryl groups was found with conarachin, which may lead to the formation of more disulfide bonds at the interfaces of the conarachin emulsion, thus forming a highly viscoelastic interfacial film and resulting in the emulsion being able to resist centrifugation. However, the centrifugal stability of the conarachin emulsions decreased to a low level after pH_2.5_-shifting or US+pH_2.5_-shifting treatments, which was consistent with the results of 4 weeks of storage.

As for arachin emulsions, both US and pH_2.5_-shifting treatments significantly improved their centrifugal stability, which may be related to the significant increases not only in the absolute potentials but also in their surface hydrophobicity and exposed sulfhydryl content. The latter structural changes are beneficial to protein cross-linking by hydrophobic interactions and disulfide bonds at oil-water interfaces. The centrifugal stability index of the arachin emulsion sharply increased from 51% to 88% after pH_2.5_-shifting treatment alone. The US+pH_2.5_-shifting treatments failed to further improve its centrifugal stability.

The physical stability of peanut protein-stabilized emulsions in the presence of salts was further investigated (Fig. 6D). Creaming occurred at different levels in all untreated and US-treated samples after storage for only 1 day. After storage for 4 weeks, untreated and US-treated conarachin emulsions showed poor stability. The addition of salts weakened the electrostatic repulsion among interfacial protein molecules due to electrostatic shielding effect. In this case, all emulsion potentials are reduced to a lower level and other protein structural properties will determine the stability of the emulsions. After pH_2.5_-shifting or US+pH_2.5_-shifting treatments, the surface hydrophobicity and exposed free sulfhydryl content of conarachin and arachin were significantly increased. These structural changes were more conducive to the interaction of proteins at the interfaces and the formation of viscoelastic interfacial films (McClements, 2015), thus improving the emulsion stability.

## 4 Conclusion

In summary, pH_2.5_-shifting was more effective in modifying peanut protein structure than US treatment. pH_2.5_-shifting rather than US treatment could partially degrade the subunits of conarachin and arachin. pH_2.5_-shifting or US+pH_2.5_-shifting treatments significantly increased the surface hydrophobicity of conarachin and arachin, which may be responsible for the enhancement of protein emulsifying activity. Both US and pH_2.5_-shifting treatments promoted conarachin aggregation and arachin disaggregation, which led to opposite changes in the ζ-potential of the emulsions stabilized by conarachin and arachin. The emulsions prepared by conarachin treated with pH_2.5_-shifting or US+pH_2.5_-shifting showed poor stability probably due to their lower absolute potentials. However, these treatments could improve the stability of conarachin-stabilized emulsions in the presence of salts. This study has advanced the understanding of the changes in the physiochemical properties of peanut proteins induced by US and pH 2.5-shifting treatments.

## CRediT authorship contribution statement

**Lin Chen:** Writing – original draft, Investigation. **Shao-Bing Zhang:** Writing – review & editing, Writing – original draft, Supervision, Funding acquisition, Conceptualization. **Shu-Yan Zhang:** Investigation, Formal analysis, Data curation.

## Declaration of competing interest

The authors declare that they have no known competing financial interests or personal relationships that could have appeared to influence the work reported in this paper.

## Data Availability

Data will be made available on request.
